# Introduction to the *RSC Advances* Emerging Investigators Series 2024

**DOI:** 10.1039/d5ra90117k

**Published:** 2025-11-06

**Authors:** Fabienne Dumoulin, Shirley Nakagaki

**Affiliations:** a Acibadem Mehmet Ali Aydinlar University Türkiye; b Universidade Federal do Paraná Brazil

## Abstract

Professor Fabienne Dumoulin and Professor Shirley Nakagaki are delighted to introduce the *RSC Advances* Emerging Investigators Series, which highlights some of the very best work of early career researchers.
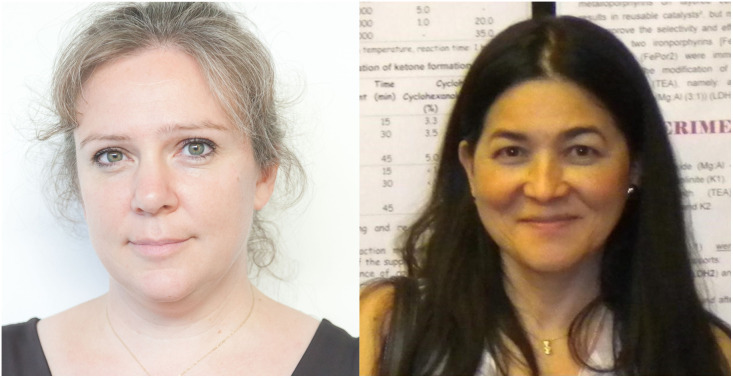

We are proud to present the fourth instalment of the *RSC Advances* Emerging Investigators Series (2024 edition). This year, we focused on interesting studies showcasing researchers' efforts to obtain information that could potentially address the United Nations Sustainable Development Goals (UN SDGs) (https://sdgs.un.org/goals). Here we present nine publications that show an advance in chemistry and support societal efforts for sustainable development.

As always, we are immensely grateful to all authors for their outstanding contributions, as well as the editors and referees for their support, which has led to another impressive edition of the *RSC Advances* Emerging Investigators Series. We hope you enjoy the excellent work carried out by young researchers.

The first piece of work is a review article entitled “Pulsed electric field technology as a promising pre-treatment for enhancing orange agro-industrial waste biorefinery” by authors R. Bocker and E. K. Silva from the Universidade Estadual de Campinas (UNICAMP), São Paulo, Brazil (https://doi.org/10.1039/d3ra07848e). This review presents a comprehensive discussion of the innovative biorefinery strategies for the valorisation of orange processing waste (OPW). Oranges are one of the most produced and consumed fruits in the world. In the processing of orange juice, 50–70% of the fresh fruit weight is converted into organic waste. Improper disposal of this residue can lead to greenhouse gas emissions, environmental pollution, and wastage of natural resources. In this way, the review focuses on the potential of employing pulsed electric field (PEF) technology as a pre-treatment to improve energy efficiency in biorefinery processes. They also discuss recent studies assessing the technical feasibility of methodologies for the extraction of phytochemical compounds, dehydration processes, and bioconversion methods. The authors conclude that the appropriate disposal of the residues mentioned is not only an environmental need but also an economic opportunity. The issues discussed in this review are in line directly and indirectly with the UN SDGs 2 (zero hunger), 9 (industry, innovation, and infrastructure), 12 (responsible consumption and production), and 13 (climate action).

The next paper, entitled “effect of a novel drying method based on supercritical carbon dioxide on the physicochemical properties of sorghum proteins”, is by the group of A. Ubeyitogullari from the University of Arkansas, Fayetteville, USA (https://doi.org/10.1039/d3ra07426a). The authors use supercritical carbon dioxide to produce sorghum protein concentrates and isolates. They demonstrated that supercritical carbon dioxide resulted in proteins with better functionalities compared to those prepared by freeze-drying techniques. This opens new avenues for obtaining functional foods, which is crucial, especially considering the need and market for proteins and the usual environmental cost of this industry. This impactful work matches SDGs 2 and 12.

In the previous Emerging Investigators Series, a group of researchers led by M. O. Alfred and E. I. Unuabonah, discussed the toxicity of dihydroxybenzenes in drinking water sources in Nigeria (https://doi.org/10.1039/d3ra04877b). Now, in the article “Prevalence and health risk evaluations of mycotoxins in drinking water sources in Nigeria” (https://doi.org/10.1039/d4ra04866k), the research group return to the problem of polluted drinking water sources in Nigeria. This time, the group, led by M. O. Omorogie and E. I. Unuabonah, focus on mycotoxins. Their recent study investigates the distribution and apparent health risks of common mycotoxins (deoxynivalenol (DON), ochratoxin A (OTA), and zearalenone (ZEN)) found in drinking water sources (groundwater, surface water, bottled water, and sachet water) in three South West Nigeria States. One of the findings of the article was that DON and ZEN were found in all 95 water samples analysed. The authors emphasise the need for a sustainable and efficient method to address the presence of these dangerous mycotoxins in Nigeria and across Africa. This work is a step towards providing people with safe drinking water, which is in line with SDG 6 (clean water and sanitation).

The development of functional organic materials is crucial for the advancement of various fields, such as optoelectronics, energy storage, sensing, and biomedicine. The next paper, from researchers based at three Argentine universities – Universidad Nacional de Río Cuarto, Universidad Nacional de Córdoba and Universidad Nacional de Mar del Plata – is called “An ambipolar PEDOT-perfluorinated porphyrin electropolymer: application as an active material in energy storage systems” (https://doi.org/10.1039/d4ra00945b). Led by M. A. Gervaldo and D. A. Heredia, the authors prepared a stable ambipolar perfluoroporphyrin-based polymeric film using electrochemical synthesis. This involved the synthesis of a novel tetra-pentafluorophenyl porphyrin covalently linked to four 3,4-ethylenedioxythiophene (EDOT) moieties. Electrochemical polymerization of the resulting monomer, EDOT-TPPF16, permits polymer synthesis and film formation. The authors show that PEDOT-TPPF16 presents pseudocapacitive behaviour, a property that makes it a promising material for energy storage devices.

Thin films and coatings based on Group 6 metals, *e.g.* tungsten (W) have garnered intense interest for applications including catalysis, lubrication, and solar energy. In line again with UN SDG 7, the article “Thermogravimetric analysis of commercial tungsten molecular precursors for vapor phase deposition processes” (https://doi.org/10.1039/d4ra07284g), investigates the use of thermogravimetric analysis (TGA) as a classification criterion for the suitability of commercially available molecular precursors for the preparation of W oxides, dichalcogenides and elemental metal films using atomic layer deposition (ALD). The author group, led by T. Jurca at the University of Central Florida, highlight several commercial precursors not yet reported for ALD growth. The results achieved provide a unified and rigorous comparison of different precursor properties that can serve as a reference map for the selection of precursors for the growth of W-based films with applications including solar energy.

Thermoresponsive materials have huge technological potential. In their article, entitled “Temperature-dependent yield stress and wall slip behaviour of thermoresponsive Pluronic F127 hydrogels” (https://doi.org/10.1039/d4ra04825c), S. N. Sangitraa and R. K. Pujala investigate the temperature-dependent dynamic yield stress of Pluronic F127 ((PEO)_100_(PPO)_65_(PEO)_100_), a well-known triblock polymer, during the sol–gel transition. The authors, from the Indian Institute of Science Education and Research (IISER), employed different strategies, such as the non-Newtonian Herschel–Bulkley (HB) model, then the Mooney method. Finally, they modified the Windhab model equation by adding slip boundary conditions to the HB equation, to demonstrate that the yield stress of these polymers follows the Boltzmann equation and increases with temperature. The authors emphasise that wall slip effects must be considered when performing rheological studies, to better evaluate the potential of such materials, which is particularly important given their use as drug carriers and delivery systems, thereby contributing to SDG 3 (good health and well-being).

Also in line with SDG 3, V. Fasano (Università degli Studi di Milano, Italy) and team achieve the first total synthesis of caerulomycin K, a natural alkaloid with antimicrobial properties. In their article, entitled “First total synthesis of caerulomycin K: a case study on selective, multiple C–H functionalizations of pyridines” (https://doi.org/10.1039/d4ra00589a), caerulomycin K is readily obtained in three steps, *via* Minisci *ortho*-arylation and *ortho*-alkylation followed by a one pot conversion into the targeted oxime. This is thanks to the recent progress in C–H activation of *N*-heterocycles. The advantage here is that highly pre-functionalised building blocks are not required – this simplifies the whole synthetic process. This paper presents pure organic chemistry with biomedical implications.

The next article, entitled “Inverse design of lateral hybrid metasurfaces structural colour: an AI approach” (https://doi.org/10.1039/d4ra04981k), is from the group of M. K. Hedayati, based at Durham University, UK. The importance and potential of chromophores and coloured materials is widely recognised, and simulation work has a huge role to play. In this work, the authors use artificial intelligence to perform mapping between metasurface parameters and colour coordinates, without the need for complex simulations. Instead of the usual method, which assigns one colour to one geometry, this single model generates multiple colours from a single geometry under varying levels of strain. This work will contribute to efficient development of active metamaterials with concrete applications.

The final paper is a multidisciplinary and international collaborative study by R. Misra (Humboldt-Universität zu Berlin, Germany) and P. K. Samanta (Gandhi Institute of Technology and Management and BITS Pilani in Hyderabad, India). The work focuses on intramolecular charge transfers occurring in pyrrolopyrrole aza-boron dipyrromethene (PPAB) push–pull dyads. The paper, entitled “Rational design and investigation of nonlinear optical response properties of pyrrolopyrrole aza-BODIPY-based novel push–pull chromophores” (https://doi.org/10.1039/d4ra02861a) shows these D–π–A molecules exhibit excellent second non-linear optical properties with high hyperpolarizability. The use of quantum chemical analysis showed sound structure–property relationships, paving the way for future applications based on NLO properties.

We are very proud to present the high-quality work carried out by these young researchers, and we are excited to see how these studies will contribute to the UN SDGs and help shape future research. We hope you enjoy the fourth edition of the *RSC Advances* Emerging Investigators Series!

If you have been inspired by the work presented in this series, and are interested in submitting to the next collection, please do contact the journal for more information. We welcome papers and reviews related to the highlighted fields and all areas of chemistry.

